# Mutant protein chemical rescue: From mechanisms to therapeutics

**DOI:** 10.1016/j.jbc.2025.108417

**Published:** 2025-03-18

**Authors:** Timothy R. O'Meara, Brad A. Palanski, Maggie Chen, Yingfeng Qiao, Philip A. Cole

**Affiliations:** 1 Division of Genetics, Department of Medicine, Brigham and Women’s Hospital, Boston, Massachusetts, USA; 2 Department of Biological Chemistry and Molecular Pharmacology, Harvard Medical School, Boston, Massachusetts, USA; 3 Department of Pharmacology and Molecular Sciences, Johns Hopkins School of Medicine, Baltimore, Maryland, USA

**Keywords:** cells, enzyme, mutation, small molecule, therapeutics

## Abstract

Chemical rescue is a technique for restoring the activity and/or structure of an engineered or naturally occurring (*e.g.*, disease-associated) mutant protein by the introduction of a “molecular crutch” that abrogates the mutation’s effect. This method was developed about 4 decades ago to facilitate mechanistic analysis of enzymes. Since then, a variety of purified proteins inactivated by site-directed mutagenesis have been successfully rescued by substrate moieties or exogenous small molecules, an approach that has continued to serve as an important tool for mechanistic enzymologists. More recently, chemical rescue has been applied to activate engineered proteins in intact biological systems for phenotypic and pathway-level analyses. There is growing interest in therapeutic applications of chemical rescue to correct protein mutations that give rise to human diseases. In this review, we first contextualize chemical rescue and discuss its utility in protein mechanistic analysis. Second, we review the advantages and caveats associated with using this approach to study protein function within biological settings. Third, we provide an overview of efforts to develop folding correctors that restore the proper function of disease-associated protein mutants. To conclude, future directions and challenges for the chemical rescue field are discussed.

During the past century, the study of protein structure and function has been a major discipline connecting the fields of chemistry and biology. Early methods for studying structure-function relationships in proteins utilized group-modifying reagents like iodoacetamide for Cys residues to probe the requirement of particular amino acids in catalysis. With the advent of modern molecular biology techniques, it became possible to engineer proteins to study the role(s) of defined amino acid residues (1). Chemical rescue, a method in which a functionally important moiety is mutated and then restored by an exogenous reagent, arose as a natural extension of these approaches. Today, in addition to serving as a tool for the mechanistic analysis of individual proteins, chemical rescue is used to study protein function and signaling in intact biological systems and for the therapeutic correction of mutations that cause human diseases (Fig. 1). In this Review, we summarize the established methods for chemical rescue and highlight examples of their influence on the fields of protein mechanism analysis, cell biology, and disease therapeutics. We also provide our viewpoint on emerging areas where chemical rescue is likely to find utility and discuss challenges that will need to be tackled in this field.

**Figure 1:**
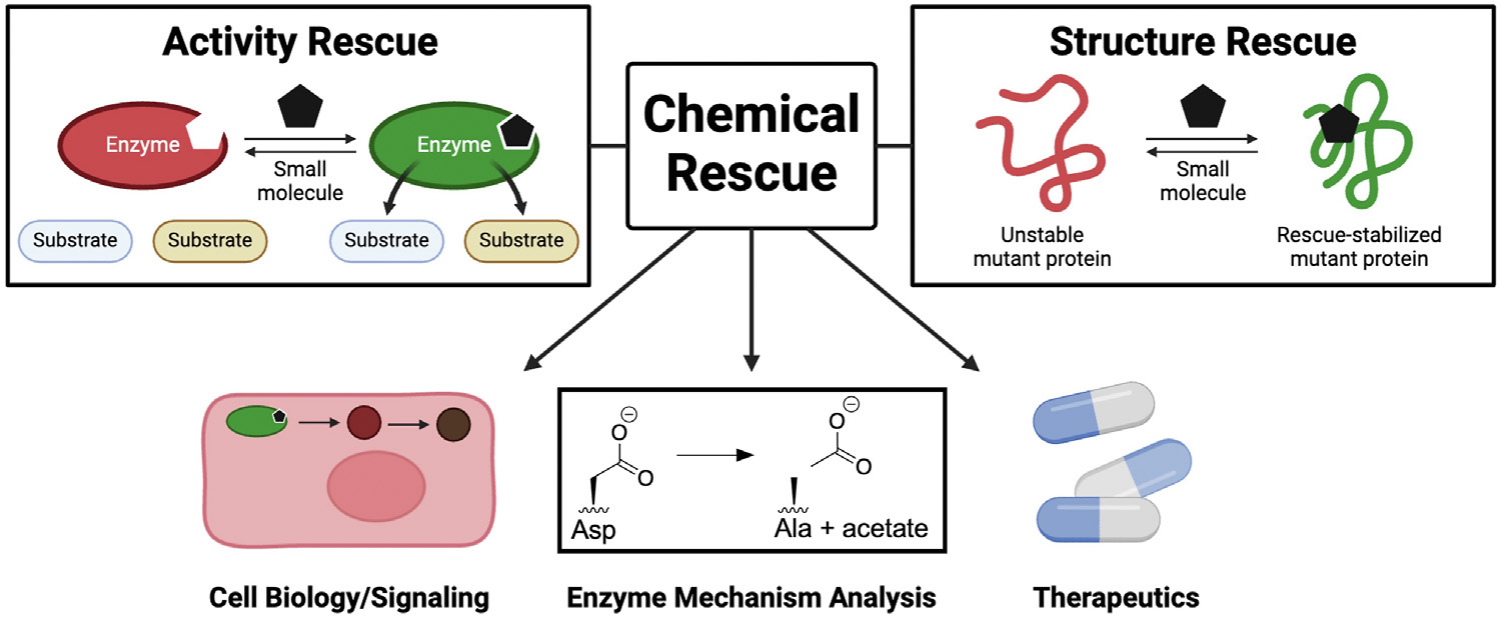
**Generalschematic/applications of chemical rescue.** Created in BioRender. O, T. (2025, https://BioRender.com/z638wk6).

## The origins, growth, and scope of chemical rescue

The earliest evidence that exogenous compounds could be introduced to compensate for the loss of functionally important enzyme moieties was gathered in the 1970s during analyses of glycogen phosphorylase (2) and peptidyl transferase (3). The historical underpinnings of chemical rescue have been reviewed elsewhere (4).

The first modern reports of chemical rescue appeared in 1987 as part of studies on the catalytic mechanism of serine proteases (5, 6). Craik et al. generated a D102N trypsin mutant that displayed a 10^4^-fold decrease in *k*_cat_, which was attributed to the loss of a hydrogen bonding network that enhances the nucleophilicity of the active site Ser. Satisfyingly, increasing pH restored mutant enzyme activity, which approached 6% of the WT level at pH 10.2. Presumably, solvent hydroxide rescued activity by providing an alternative reaction mechanism (5, 6). Contemporaneously, Carter and Wells found that mutating His64 in subtilisin’s catalytic triad to Ala decreased the catalytic efficiency (*k*_cat_/*K*_M_) by six orders of magnitude (7). However, proteolytic activity improved 400-fold when a His residue was included at the P2 position of the peptide substrate. This result demonstrated that an exogenous moiety introduced on the substrate could compensate for an enzyme mutation in a process referred to as “substrate-assisted catalysis” (7). In 1989, Toney and Kirsch reported that the activity of aspartate aminotransferase rendered catalytically dead by a K258A mutation could be restored by a variety of exogenous small molecule amines (8). By varying their molecular size and p*K*_a_, it was demonstrated that systematic structural-activity relationships using physical organic chemistry methods such as Brønsted analysis could be utilized to study enzymatic catalysis (8). Brønsted analysis is a type of linear free energy relationship in which the log of the rate of a reaction is plotted *versus* a chemical parameter (such as p*K*_a_) of a series of structurally related substrates or catalysts. When the relationship appears linear, the substrates or catalysts operate *via* the same reaction mechanism, and the slope of the line (Brønsted slope) can be determined (see W. P. Jencks, Catalysis in Chemistry and Enzymology, 1987, Dover Publications) (9). In the Toney and Kirsch study (8), the term “chemical rescue” made its debut, and a door was opened for its widespread application to the study of enzyme mechanisms, examples of which have now been reported for a wide range of enzymes in >100 publications.

The chemical rescue technique that had been extensively used *in vitro* during the prior decade began to be applied to living systems in the early 2000s. Schultz et al. showed that point mutations severely compromised the binding affinity of the human growth hormone for its extracellular receptor, but the addition of benzimidazole ligands to engineered mutant cell lines was sufficient to restore human growth hormone signaling (10). Meanwhile, our group demonstrated that protein tyrosine kinases (PTKs) made catalytically inert by active site point mutations could be reactivated by the addition of exogenous imidazole (11, 12, 13). The cell permeability and relatively low toxicity of imidazole made it a successful rescue agent for mutant PTK activity in living cells, which facilitated further phenotypic analyses of PTKs and the identification of novel substrates (11, 12, 13).

Moving beyond chemical rescue as a tool for understanding enzymes and cellular pathways and phenotypes, molecules have been identified that can rescue the structure and/or activity of disease-associated proteins, some of which are already in use as approved therapeutics. Today, interest in expanding chemical rescue methods to correct the deleterious effects of protein mutations in human diseases continues to grow.

## How to define chemical rescue?

Today, a wide variety of methods, such as the incorporation of bioorthogonally cleavable non-natural amino acid “cages” (14) and the introduction of small peptide inserts that abrogate enzymatic activity in the absence of stabilizing ligands, have been reported (15), raising the question of how to define chemical rescue. For this review, we use two broadly inclusive criteria. First, chemical rescue in this review is considered for proteins that are inactivated by mutation, typically through the replacement of an amino acid required for catalysis or folding. These mutations may occur naturally, as in diseases, or result from protein engineering. We will not discuss strategies for the overactivation of native proteins or enzymes that naturally rely on substrate functional groups for catalysis, as these topics have been reviewed previously (16, 17). Second, the rescue agent must act on the protein of interest directly. Thus, we do not consider transcriptional modulation of protein expression to be *bona fide* chemical rescue, even if activity or structure is ultimately affected.

## Chemical rescue for enzyme mechanistic analysis

The structural and electronic features of chemical rescue agents can be precisely varied, thereby allowing tools available to physical organic chemists to be applied to the study of enzymatic reaction mechanisms. For example, Brønsted linear free energy analysis, which is useful in delineating transition state structure, requires that the reaction rate constant be determined as a function of the acid or base ionization constant. However, with the limited repertoire of 20 canonical amino acids, it is impossible to manipulate the ionization constant of a catalytic residue with enough precision to facilitate a proper Brønsted analysis by site-directed mutagenesis alone. Chemical rescue overcomes this limitation by allowing analysis of the reaction rate in the presence of a wide variety of small molecules.

To date, chemical rescue techniques have been reported for at least 10 of the 20 canonical amino acids. In the most common scenario, a catalytically important residue is mutated to a smaller, chemically inert residue such as Gly or Ala, which is then complemented by the addition of a molecule that has stereoelectronic properties similar to the original residue and reversibly binds in the “cavity” created by the mutation. However, as summarized in Table 1, a wide and sometimes surprising variety of combinations of mutations and exogenous rescuing molecules have been utilized. In this section, we review selected examples of the use of chemical rescue to establish structure-activity relationships, delineate reaction mechanisms, and enable physical organic chemistry analyses of the enzymes. We emphasize studies whose conclusions would have been difficult to reach without chemical rescue and point out the caveats and limitations associated with the interpretation of the results. For organizational purposes, the examples are grouped by the identity of the mutated amino acid.

**Table 1:** Summary of chemical rescue techniques that have been reported

Original amino acid	Reported mutations	Common rescue agents	Example proteins
Arg	Gly, Ala, Lys, Gln, Trp, Cys, Met	Imidazoles, guanidines, primary amines	Rat pancreatic carboxypeptidase A (18), ornithine transcarbamylase (19), glutamine synthetase (20), asparagine synthetase (20), S-mandelate dehydrogenase (22), IMP dehydrogenase (23), trehalose phosphorylase (24), protein tyrosine kinases including Src (12), Csk (13), and Abl (25)
Asp	Ala, Asn, Gly, Gln, Ser	H^+^, azide, formate, fluoride, acetate	Trypsin (26), glutamate dehydrogenase (27), β-class carbonic anhydrase (28), trans-sialidase (29), α-L-fucosidase (30), hexosaminidase (31), glucosylceramidase (32), core fucosidase (33), β-*N*-acetylglucosaminidase (34), α-galactosidase (35), NEDD4 E3 ligase (36)
Cys	Ser, Ala, Gly	2-mercaptoethanol	PsaC (40, 41)
Glu	Gly, Ala, Gln	Azide, acetate, formate	1,3-1,4-β-glucanase (42), α3-Galactosyltransferase (43), β-xylosidase B (44), dextranase (45), β-galactosidase (50)
Gln	Leu	GTP analogs	G_s_ α subunit (G protein) (53)
His	Ala, Gly, Asp, Gln, Asn, Ser	Imidazoles	Subtilisin (7), thiamin pyrimidine synthase (55), SpyCas9 (56), nucleoside diphosphate kinase (57)
Leu	Ala, Gly	Pyrene base	Uracil-DNA glycosylase (58)
Lys	Ala, Cys, Met, Arg, Thr, Glu, Phe	Primary amines, short-chain carboxylic acids	Leucine dehydrogenase (59), rhodopsin (60), tryptophan synthase (61), alanine racemase (62), isochorismate-pyruvate lyase (63), aspartate aminotransferase (8, 64), pyruvate kinase (65), allantoinase (68)
Trp	Gly, Ala	Indoles	β-glycosidase (69); +5 GFP, β-glucuronidase, YeaZ, Orn, TadA (70)
Tyr	Gly, Phe	Phenol, acetate, bromide	Ketosteroid isomerase (71), xylose reductase (72), TbtD pyridine synthase (73)

### Arginine

In 1992, Phillips et al. reported the first chemical rescue of an Arg residue when probing the role of Arg127 in rat pancreatic carboxypeptidase A (18). This protease cleaves the C-terminal side of a peptide bond *via* a Zn^2+^-promoted nucleophilic water attack. R127A and R127M mutations decreased *k*_cat_ by four orders of magnitude with little effect on *K*_M_. Guanidine, methylguanidine, and ethylguanidine increased the *k*_cat_ of the R127A mutant by up to 100-fold. Guanidine restored the binding of a transition state analog but not a ground-state inhibitor in the R127A mutant, indicating that the primary role of Arg127 is transition state stabilization rather than substrate binding. Guanidine, but not its bulkier alkylated derivatives, rescued the R127M mutant, presumably due to steric hindrance. Importantly, this observation suggested that rescue occurred through direct substitution for Arg127 in the active site.

Ornithine transcarbamylase catalyzes the production of L-citrulline and phosphate from carbamyl phosphate and L-ornithine. Mutation of a conserved Arg (residue 57 in *Escherichia coli*) severely decreased *k*_cat_ (19). The activity of an R57G variant was restored by a variety of exogenous guanidines but not by primary amines, pyridine, or arginine itself (19). Guanidinium hydrochloride was the most potent rescue agent, stimulating *k*_cat_ by 2000-fold to within 10% of the WT enzyme. This molecule displayed a half-maximal rescue concentration (*K*_r_) of 22 mM, while other rescue agents displayed *K*_r_ values two orders of magnitude higher, suggesting that guanidinium hydrochloride can make specific binding interactions in the active site cavity. Structure-activity relationship studies using compounds with various molecular volumes and p*K*_a_ values enabled a detailed investigation of the catalytic role of Arg57. A modified Brønsted analysis accounting for both base strength and molecular volume yielded a Brønsted slope of 0.67 (β = 0.67), arguing for a partial positive charge buildup in the transition state. NMR studies supported the suggestion that guanidines, and by extension Arg57, function to stabilize charge development at the transition state of the transcarbamylation reaction. Interestingly, multiple inhibitor experiments revealed that the kinetic mechanism of rescued R57G ornithine transcarbamylase is random, whereas the WT enzyme is ordered sequential. The basis for this unanticipated mechanistic change was not fully addressed.

**Figure 2:**
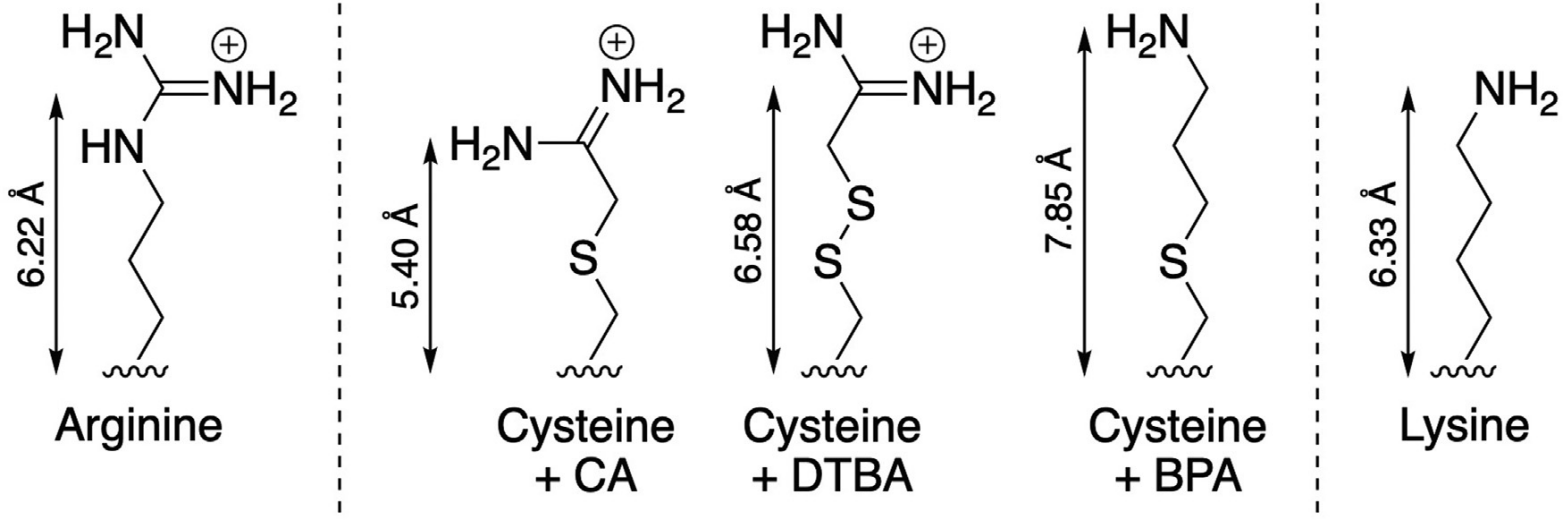
**Chemical rescue of an Arg residue by modification with covalent Cys reactive reagents.** Adapted from Dhalla *et al.* (20). Modifying agents: BPA, 3-bromopropylamine; CA, 2-chloroacetamidine; DTBA, 2,2′-dithiobis(acetamidine).

The amino acids Asn and Gln are biosynthesized from Asp and Glu in the presence of an ammonia source and ATP by glutamine synthetase (GS) and asparagine synthetase (AS). GS and AS utilize different catalytic cycles but both possess active site Arg residues (20). Dhalla et al. generated R339C and R359C mutants of *E. coli* GS and found that the enzymes displayed a 40–100-fold increase in *K*_M_ for both ATP and Glu and a similar fold decrease in *k*_cat_ (20). The nucleophilic Cys residue in the mutants were covalently modified by 2-chloroacetamidine (2-CA), 2,2′-dithiobis(acetamidine) (DTBA), or 3-bromopropylamine (Fig. 2). Importantly, control experiments demonstrated that these agents modified only the active site Cys339 and Cys359 and that the reactions reached completion. For both mutant enzymes, *k*_cat_, *K*_M_,_ATP_, and *K*_M_,_Glu_ were restored to nearly their WT values by modification with 2-CA. In contrast, DTBA did not efficiently rescue the *k*_cat_ of the R359C mutant, and 3-bromopropylamine could not reactivate this enzyme. The fact that only 2-CA and DTBA were able to restore activity to the R359C mutant suggested that Arg359 interacts with substrates in a bidentate fashion. Moreover, a comparison of the rescued *k*_cat_ and *K*_M_ values after modification with 2-CA and DTBA indicated that their side chain length, which differs by only 1.1 Å, substantially affected substrate binding and catalysis. This effect was most pronounced with R359C GS, as *k*_cat_ was restored to 70% of the value of the WT enzyme by 2-CA but was hardly affected by DTBA. In a separate study, a more conventional chemical rescue strategy utilizing an Arg→Ala mutation was employed to probe the role of Arg325 in *E. coli* AS B (21). Addition of 50 mM guanidinium hydrochloride restored specific activity to ∼15% that of the WT enzyme. *K*_M_ values for glutamine (the ammonia donor) and ATP in the rescued enzyme were identical to the WT protein, while the rescue of *K*_M_ for aspartate could not be quantified due to an inability to reach saturating concentrations. Taken together with structural evidence, these results suggested that R325A plays a role in aspartate substrate binding and stabilization of the transition state leading to the β-aspartyl-AMP intermediate.

S-mandelate dehydrogenase catalyzes the oxidation of the α-hydroxy group of S-mandelate using an FMN cofactor. Chemical rescue facilitated detailed thermodynamic and kinetic studies on the roles of a conserved active site Arg277 in this enzyme from *Pseudomonas putida* (22). An R277G mutation decreased *k*_cat_ and increased *K*_M_ by approximately 400-fold each. SAR analysis revealed that substituted guanidine and imidazole compounds restored activity. 1-methylguanidine was the most potent rescue agent, giving half-maximal rescue at 10 mM (*K*_r_ ≈ 10 mM) and stimulating *k*_cat_ and *K*_M_ to within 1.5-fold and 30-fold of the WT values, respectively. Interestingly, despite a conservative R277K mutant displaying a similar *k*_cat_ and *K*_M_ to the R277G protein rescued with 1-methylguanidine, the primary substrate kinetic isotope effect on *k*_cat_, ^D^*k*_cat_, was markedly increased in the R277K mutant but identical to the WT protein in the rescued R277G mutant. This result suggested that chemical rescue faithfully preserved the rate of the α-carbon-hydrogen bond breaking step. The differences in substrate binding energy (ΔΔG_S_), the activation energy associated with *k*_cat_ (ΔΔG^‡^), and the activation energy associated with *k*_cat_/*K*_M_ (ΔΔG_T_^‡^) were calculated for the WT, mutant, and rescued proteins. This analysis revealed that the positive charge of Arg277 is primarily responsible for transition state stabilization, while the hydrogen-bonding capacity of this residue contributes significantly to both transition state stabilization and substrate binding. Further experiments suggested the requirement for a hydrogen bond donor at the N-1 position of the rescuing molecule, as 1,1-dimethylguanidine and 1-methylimidazole were not effective rescue agents.

Guillén Schlippe and Hedstrom used chemical rescue to study the role of an active site Arg in inosine 5′-monophosphate dehydrogenase, which catalyzes the oxidation of inosine 5′-monophosphate to xanthosine 5′-monophosphate (XMP) using an NAD^+^ cofactor (23). The reaction mechanism involves a conformational change in the enzyme after the release of the NADH product, followed by the attack of a water molecule on an E-XMP∗ intermediate. Prior studies suggesting that this conformational change positioned Arg418 to deprotonate and activate water for nucleophilic attack were controversial since the p*K*_a_ of free Arg is ∼13. Replacement of Arg418 with Ala reduced *k*_cat_ over 500-fold. Activity was restored to 20% of the WT value with several guanidine derivatives, which displayed *K*_r_ values from 40 to 390 mM. The apparent saturation observed with some of these molecules was attributed to competition between increased occupancy of the binding site and denaturation of conformationally important structural elements. Notably, the efficiency of the rescue reaction increased with pH, providing support for the role of Arg as a general base. Brønsted analysis with guanidine derivatives yielded β ∼ 1, suggesting that the proton from water is almost completely transferred to Arg418 in the transition state. Taken together with solvent isotope effect experiments, chemical rescue supported a concerted reaction mechanism in which Arg418 acts as a general base to deprotonate water at the same time as it attacks the E-XMP∗ intermediate.

Trehalose phosphorylase catalyzes the phosphorolysis of α,α-trehalose into α-D-glucose 1-phosphate and α-D-glucose using a catalytic mechanism similar to many glycosyltransferases that contain an RXXXXK active site motif (24). Although the structures of many of these glycosyltransferases had been solved, the relative contributions of the active site Arg and Lys residues to catalysis remained unclear for many years. Goedl and Nidetzky found that the R507A mutant of this enzyme displayed a 10^5^-fold decrease in *k*_cat_ and a 130-fold increase in *K*_M_ for phosphate (24). Enzymatic activity was enhanced up to 50-fold in the presence of guanidine and various derivatives, but no clear relationship between rescue, p*K*_a_, and/or molecular size could be determined (24). Nonetheless, combined with additional chemical studies of Lys512 (discussed in the “Lysine” section below), the authors were able to determine that the primary role of Arg507 is transition state stabilization and not substrate binding.

**Figure 3:**
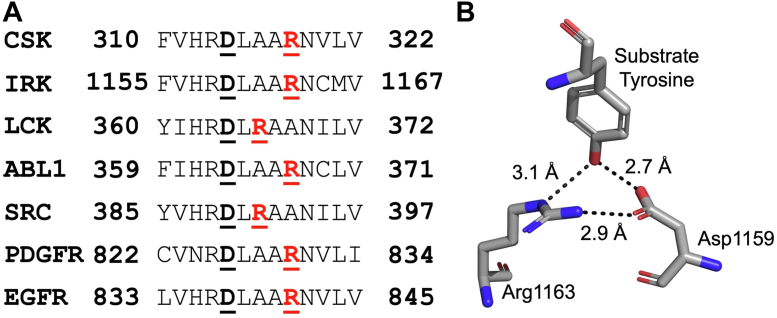
**Chemical rescue of PTKs.***A*, PTK active site alignment (13). The highlighted Asp (D, *black*) is the highly conserved catalytic base in protein kinases and the highlighted Arg (R, *red*) is conserved in one of the two positions in tyrosine kinases. In Src family members, it is two residues downstream of the catalytic base and in non-Src family members, it is four residues downstream. *B*, PTK structural arrangement of the conserved Asp and Arg in complex with a substrate Tyr, adapted from Hubbard (PDB: 1IR3) (126).

PTKs catalyze the transfer of the γ-phosphate of ATP to protein tyrosine residues through a dissociative transition state. Within their active site, these enzymes possess a conserved Arg residue that is two or four residues downstream of an absolutely conserved catalytic Asp base (11, 12, 13). Structural studies show that the side chain of the Asp makes a hydrogen bond to the tyrosine hydroxyl, while the Arg side chain makes apparent hydrogen bonds to this Asp side chain and the substrate phenol oxygen (Fig. 3). These bonds appear to provide a scaffold that aligns the reactants. Studies on three PTKs (Csk, Src, and Abl), have demonstrated that in each case, Arg→Ala mutants in the catalytic loop reduce enzyme activity by 300 to 3000 fold, which is mainly attributable to a drop in *k*_cat_ (11, 12, 13, 25). Guanidinium compounds can rescue PTK activity. Interestingly, however, in all cases studied so far, imidazoles are more potent activator, restoring up to half of the activity of the WT protein at low millimolar concentrations in Src and Abl (11, 12, 13, 25). Crystallographic and pH-dependence studies in Abl have elucidated the binding mode of imidazole and indicate that the imidazolium ion is a more potent rescue agent than neutral imidazole (11, 12, 13, 25). This fortuitous finding that imidazole, a cell-permeable and relatively nontoxic molecule, is a potent reagent for PTK rescue has been taken advantage of to study PTK signaling in intact cells. These studies are discussed later.

### Aspartate

When chemical rescue was first applied to study serine proteases, Asp was the lone member of the catalytic triad whose function was still somewhat controversial (5, 6). Solvent hydroxide was found to partially rescue a trypsin D102N mutant, suggesting the key function of Asp102 in the catalytic triad was to enhance the nucleophilicity of the active site Ser. This mechanism was supported by crystallographic findings showing that Asp indirectly enhanced Ser nucleophilicity by stabilizing the His tautomer needed to enhance Ser deprotonation (5, 6). Notably, chemical rescue was later applied to study the role of trypsin Asp189, which is not situated at the active site but rather at the specificity pocket (26). This Asp was suspected to play an electrostatic role in setting trypsin’s preference for Arg and Lys residues at the P1 position of its substrates. In the crystal structure of the D189S mutant, an acetate ion from the crystallization solvent was visible at the base of the P1 specificity pocket, suggesting that acetate might rescue the activity of this mutant (26). Indeed, it was found that exogenous acetate, and to a lesser extent formate, could enhance the activity of D189S trypsin (26). Interestingly, rescue was 10-fold more pronounced for substrates bearing Arg at the P1 position than for those with Lys (26). Further comparison of rescue with various model substrates revealed that only the amidase (and not esterase) activity of trypsin could be recovered upon acetate addition (26). While acylation is the rate-limiting step in the amidase reaction, deacylation is rate limiting in the esterase reaction. Taken together, the data suggested a model in which Asp189 played a dual role in supporting substrate binding (as reflected in *K*_M_) and catalysis by accurately positioning the scissile bond for cleavage (reflected in *k*_2_, the rate constant for acylation).

Glutamate dehydrogenases (GDHs) possess a critical active site Asp situated in a position where it could receive a proton from the α-amino group of the α-ketoglutarate substrate (27). Mutation of Asp156 in clostridial GDH reduced its oxidative deamination activity by 100,000-fold and the reverse reductive amination reaction by 1000-fold (27). Hayden et al. attempted to rescue the D156S mutant with small molecules, including formates, acetates, and halides (27). Only fluoride was active, suggesting that rescue involved this ion binding to a specific site rather than a general effect caused by ionic strength changes (27). Interestingly, a seconds-to-minutes lag phase was observed prior to rescue, but circular dichroism did not reveal any significant perturbations to GDH’s structure upon fluoride addition, arguing against a large shift in enzyme conformation. Fluoride displayed a *K*_r_ of 200 mM for the forward reaction and 40 mM for the reverse reaction. Notably, although the reverse reaction had a substantially lower *K*_r_, the maximum extent of rescue (a 4-fold increase over baseline) was dramatically lower than the extent of rescue for the forward reaction (a 1000-fold increase). Because F^-^ is a poor proton acceptor at neutral pH, the authors suggest that Asp156 likely provides a fixed charge at the active site. However, acid/base effects cannot be conclusively ruled out as the p*K*_a_ of Asp may vary depending on the active site microenvironment.

β-class carbonic anhydrases use a two-step mechanism to catalyze the conversion of carbon dioxide to bicarbonate (28). Water is activated by an enzyme-bound Zn^2+^ ion to attack carbon dioxide and then proton transfer steps regenerate carbonic anhydrase. In this enzyme family, Asp34 and Arg36 are the only absolutely conserved residues aside from those directly involved in Zn^2+^ coordination. Upon mutation of Asp34 to Ala, a modest decrease in *k*_cat_ but no effect on *k*_cat_/*K*_M_ was observed (28). Consistent with this behavior, imidazole rescued this defect in *k*_cat_ without affecting *k*_cat_/*K*_M_. Since the *k*_cat_/*K*_M_ parameter reflects the kinetic properties of an enzyme before the first irreversible step, the combined mutational and rescue experiments revealed that Asp34 participated in proton transfer steps to regenerate the active enzyme but not in the initial CO_2_ hydration step (28).

**Figure 4:**
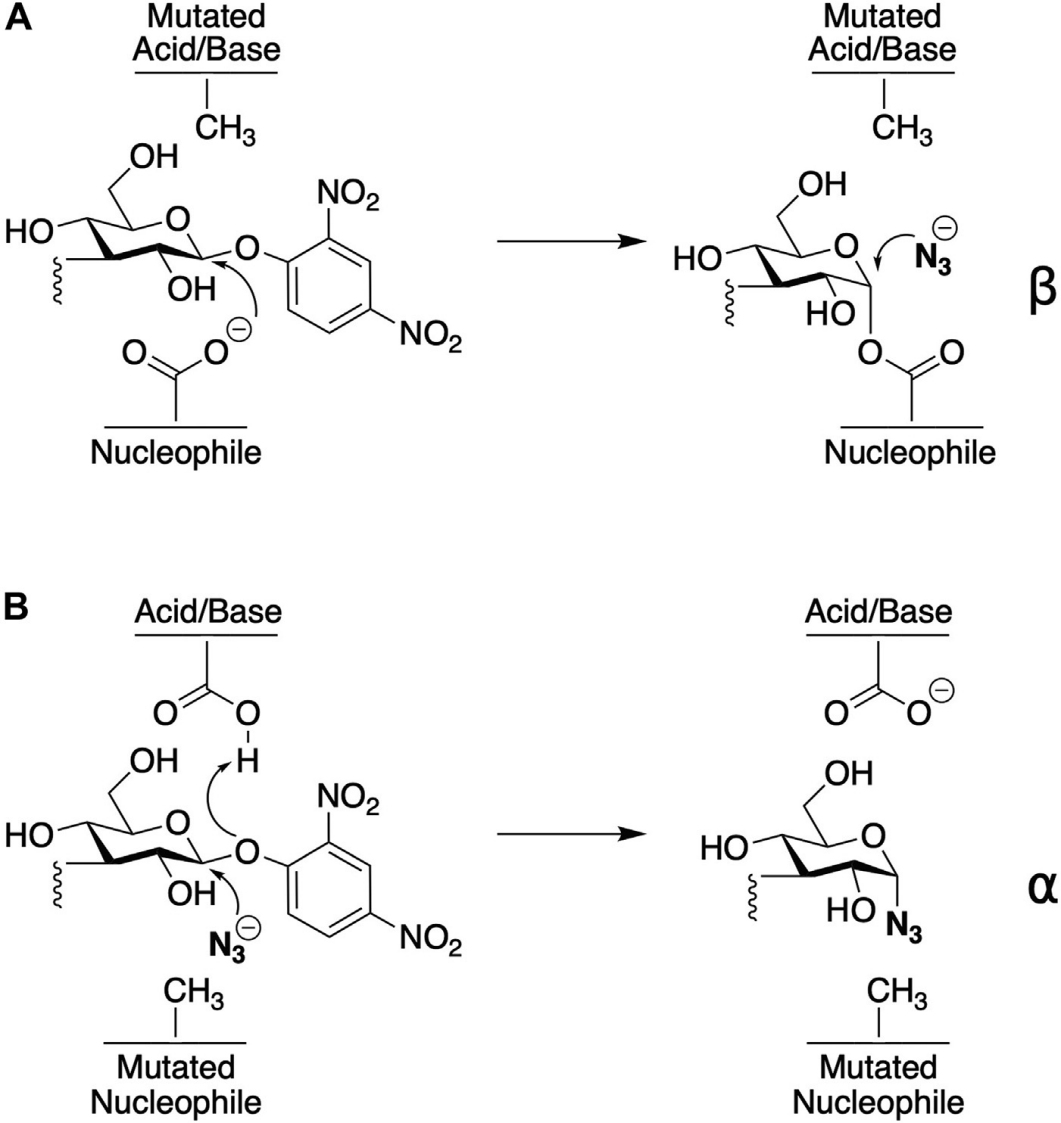
**Mechanistic analysis of glycosidases via chemical rescue.** (*A*) Acidic/basic or (*B*) nucleophilic roles of conserved Asp residues in glycosidases can be elucidated by observing glycosidic bond stereochemistry after chemical rescue.

Chemical rescue has served as a particularly critical tool for delineating reaction mechanisms in the study of glycosidase and glycosyltransferase enzymes (29). Many anomer-retaining glycosidases contain two conserved Asp and/or Glu residues, one of which functions as the active site nucleophile and the other of which acts as a general acid/base. Even when the structure of these enzymes has been solved, it can be difficult to infer which acidic amino acid plays which role. For several glycosidases/glycosyltransferases, azide has been shown to efficiently rescue enzyme activity that is lost upon mutation of Asp/Glu residues to Gly or Ala by acting as an alternative nucleophile to water, resulting in the formation of an azidosugar (29, 30, 31). If, upon isolation, the azidosugar product has the same configuration of the anomeric carbon as the substrate, then it can be inferred that the mutated Asp/Glu functioned as a general base. In contrast, if the product displays an inversion of configuration, then the mutated residue was the catalytic nucleophile (Fig. 4). This strategy has been successfully applied to elucidate the catalytic mechanisms of many glycosidase enzymes containing active site Asp residues, including *Trypanosoma cruzi* trans-sialidase, *Sulfolobus solfataricus* α-L-fucosidase, *Streptomyces plicatus* hexosaminidase, *Paenibacillus* sp. glucosylceramidase, *Elizabethkingia meningoseptica* core fucosidase, and *Vibrio harveyi* GH20 *β*-*N*-acetylglucosaminidase (29, 30, 31, 32, 33, 34). A similar approach has been applied to *Bacteroides thetaiotaomicron* glycoside hydrolase family (GH) 97 retaining α-galactosidase (BtGH97b) to enable the synthesis of α-galactosyl oligosaccharides using α-galactosyl fluoride (α-GalF) and β-GalN3 as substrates by rescuing catalytically inactive D415G BtGH97b with azide or formate (35).

Recent studies on ubiquitin E3 ligases have also used chemical rescue of an Asp mutant to assign a role for a terminal carboxylate proton transfer in the catalytic mechanism (36). These reactions involve the transfer of a ubiquitin thioester from the catalytic Cys of NEDD4, a HECT domain E3 ligase, to the sidechain of Lys in protein substrates. There have been differing views about whether the thioester aminolysis that occurs in E3 ligase reactions requires a catalytic base (37, 38, 39). Mutation of Asp900 into Ala in NEDD4, a HECT E3 ligase, substantially reduced the enzyme’s Lys ubiquitin transfer activity. Complementing the D900A mutant with acetate ions and a series of analogs revealed a Brønsted slope of 0.4, which, along with a solvent kinetic isotope, suggests that the carboxylate group is directly involved in proton transfer (36). Such functions could involve either assisting in the deprotonation of the Lys ammonium group or serving as a general acid to protonate the departing sulfur of the catalytic Cys. An especially interesting aspect of HECT E3 ligase catalysis is that some members of the family, like E6AP, lack an Asp or Glu on their C-termini and instead use their terminal backbone carboxylate for this function. When the terminal carboxylate was replaced with a carboxamide through protein semisynthesis, a steep decline in catalysis was observed (36). Other HECT family members like yeast RSP5 containing a terminal Glu can employ either the sidechain or backbone carboxylate and may be evolutionary intermediates (36).

### Cysteine

Cys plays diverse roles in proteins, including stabilizing structure and regulating activity through disulfide bonding, assisting in catalysis through acid/base and covalent chemistry, and binding to cofactors. Only the lattermost of these functions has been rescued chemically. Certain Cys to Ala mutants of PsaC, a ferredoxin-like protein found in Photosystem I, were found to retain the ability to bind [4Fe-4S] clusters despite structural and biochemical evidence that the mutated Cys served as prosthetic group ligands (40). An explanation put forth for this surprising finding was that free 2-mercaptoethanol used in PsaC purification and enzymatic assays may have substituted for the mutated Cys (40). Direct evidence was gathered that exogenous thiolates could serve as [4Fe-4S] cluster ligands in C13G PsaC by ^19^F NMR, as fluorinated thiol-donating ligands display substantial changes in their spectra upon binding to paramagnetic clusters (41). Despite this interesting initial report of using external thiols with defined steric and electronic properties to substitute for Cys ligands, this approach has not yet been reported for other proteins.

### Glutamate

As mentioned in the aspartate section above, glutamate rescue has often been performed with azide to determine whether a specific glutamate residue in an enzyme serves as the catalytic nucleophile or as a general acid/base. Such efforts have facilitated mechanistic understandings of enzymes such as *Bacillus licheniformis* 1,3-1,4-β-glucanase (42), α3-galactosyltransferase (43), and *Bifidobacterium adolescentis* β-xylosidase B (44). In the study of *Paenibacillus* sp. dextranase, formate also appeared effective at rescuing glutamate mutants, albeit at a high concentration of 2.4 M (45). Mechanistic understandings of glycosidase and glycosyltransferase enzymes derived from chemical rescue studies also laid the foundation for the development of novel approaches to synthesize monoglycosyl azides, including α-D-glucopyranosyl, β-L-fucopyranosyl, and rutinosyl α-azides (46, 47, 48, 49). In addition, the synthesis of various oligosaccharides was made possible by rescuing E361G-mutant *Alicyclobacillus acidocaldarius* β-galactosidase with azide or formate, which resulted in a 177-fold enhancement in transglycosylation activity over the WT enzyme (50).

### Glutamine

Mutation of Gln227 in the heterotrimeric G-protein G_sα_ subunit has been associated with human pituitary and endocrine tumors (51, 52). Biochemical analyses of Q227L G_α_ suggested that this mutation caused a GTPase catalytic deficiency that could be rescued to nearly WT activity by employing a GTP analog, 3,4-diamino-benzophenone phosphonoamidate-GTP (DABP-GTP), as a substrate (53). This is an example of substrate-assisted catalysis, wherein the substrate contains a crucial functional group absent in the mutant.

### Histidine

As discussed, the protease subtilisin with a H64A mutation in its catalytic triad can be rescued by substrate-assisted catalysis in which the substrate carries a His residue (7). Follow-up studies by Carter and Wells compared chemical rescue by free imidazole and showed that the catalytic activity can be partially restored with the small molecule, albeit to a much lesser degree than with a His-containing substrate (54). Detailed analysis showed that the His residue is most important in covalent ester intermediate formation involving the active site nucleophilic Ser, with a smaller effect on deacylation.

Thiamin pyrimidine synthase THI5P from the yeast *Candida albicans* is a single-turnover metabolic enzyme that catalyzes the conversion of the cofactor PLP and a histidine residue to form thiamin pyrimidine in the presence of Fe(II) and oxygen (55). To gain insight into the mechanism underlying this conversion, Begley et al. sought to identify and isolate several key reaction intermediates, including a species in which His66 of thiamin pyrimidine synthase covalently attaches to an oxidatively dearomatized PLP derivative. Treatment of H66G thiamin pyrimidine synthase with free imidazole facilitated the isolation and characterization of several proposed reaction intermediates (55).

*Streptococcus pyogenes* Cas9 (SpyCas9) is a bacterial nuclease that has been utilized for CRISPR gene editing. The SpyCas9 HNH and RuvC nuclease domains each contain a key histidine necessary for catalysis (56). H840A and H840G mutations in the HNH domain dramatically reduced SpyCas9 nuclease activity, and 100 mM imidazole substantially rescued the *in vitro* activity of both H840 mutants. Further H840A and H840G rescue experiments demonstrated a positive correlation between increasing p*K*_a_ of imidazole derivatives and SpyCas9 cleavage efficiency, suggesting that H840 functions as a general base in catalysis (56).

**Figure 5:**
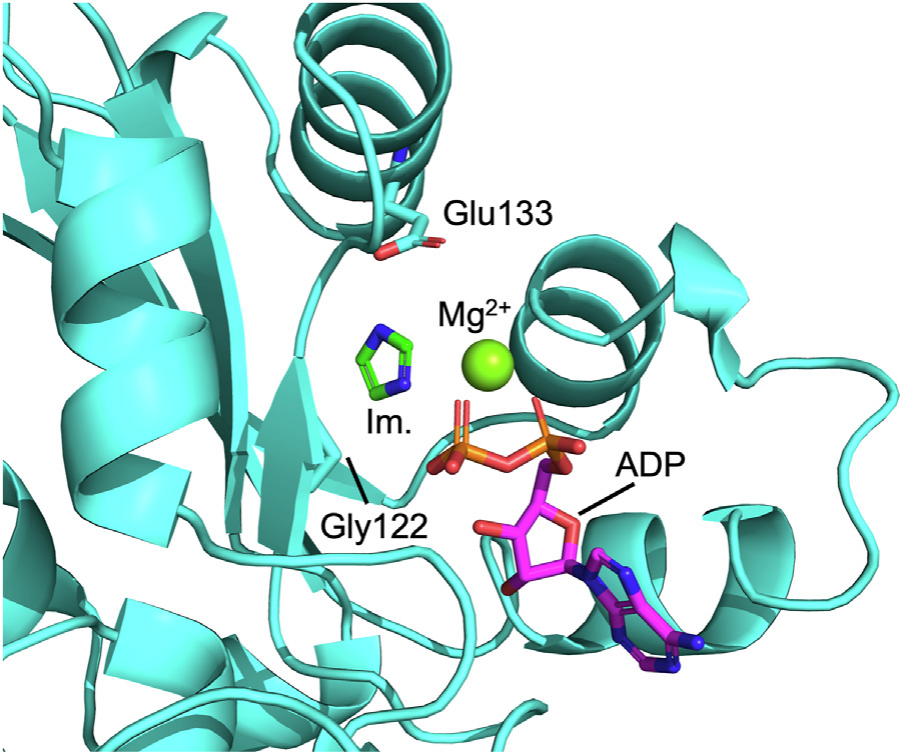
**Structural model of NDPK rescue by imidazole.** Crystal structures of WT (PDB: 1KDN) and H122G (PDB: 1B4S) NDPK bound to ADP, Mg^2+^, and AlF_3_ (WT) or P_i_ (H122G) were superimposed, and the H122G structure is shown (57, 127). Imidazole (Im., in *green*) was modeled from the His side chain of the WT enzyme. Adapted from Admiraal *et al.* (57).

*Dictyostelium discoideum* nucleoside diphosphate kinase (NDPK) is an enzyme that catalyzes the interconversion of nucleoside diphosphates and NTPs. NDPK transfers the terminal phosphoryl group from the NTP substrate onto the nucleophilic histidine His122 and can then transfer this group from His122 onto another nucleoside diphosphate to generate NTP (57). Mutation of His122 to Gly caused a 2000-fold reduction in *k*_cat_/*K*_M,ATP_ relative to the WT enzyme, while imidazole bound to H122G NDPK with an estimated *K*_d_ of 250 mM and rescued *k*_cat_/*K*_M,ATP_ by 40-fold to only 50-fold below that of the WT enzyme. In assays utilizing ATPγS in the place of ATP, the *k*_cat_/*K*_M,ATPγS_ for unrescued H122G NDPK was only 200-fold below WT, while the imidazole-rescued H122G enzyme had a *k*_cat_/*K*_M,ATPγS_ 100-fold higher than the unrescued condition and only 2-fold below WT (57). These results suggest that covalent attachment of the His nucleophile provides a substantial catalytic advantage, while the bulky sulfur disrupted the positioning of His122 to a much greater extent than imidazole. Additional rescue experiments assessing the effects of p*K*_a_ on H122G rescue by small amines yielded a Brønsted slope of 0.16 (β = 0.16), suggesting that the enzyme employs a dissociative transition state (57). An alignment of WT and H122G NDPK crystal structures further demonstrated that the H122G mutation has relatively little effect on the overall protein shape, including conservation of the structure around Glu133, which positions His122 for catalysis *via* hydrogen bonding interactions and likely plays a similar role in positioning exogenous imidazole for catalysis (Fig. 5) (57).

### Leucine

Bearing no charged or polar functional groups, leucine is the simplest amino acid for which chemical rescue has been reported. All uracil-DNA glycosylases from bacteria to mammals contain an absolutely conserved Leu. These enzymes catalyze the first step of DNA base excision repair by expelling 2-deoxyuridine from the DNA helix and hydrolyzing its N-glycosidic bond. In human uracil-DNA glycosylase, Leu191 was mutated to Gly and Ala to probe its suspected role in pushing through the DNA minor groove to expel uracil from the major groove (58). Both mutant enzymes displayed moderately impaired catalysis of uracil excision (*k*_cat_/*K*_M_ decreased 10- to 500-fold depending on the substrate). Incorporating a bulky pyrene base opposite the uracil to be excised restored activity partially for the Gly mutant and completely for the Ala mutant, suggesting that pyrene could not completely fill the space generated in the active site of the less bulky Gly mutant (58). Through kinetic rescue studies with various single-stranded, double-stranded, and nonhydrolyzable DNA substrates along with crystallographic data, a model was formed to argue that Leu191 primarily functions to “plug” the space filled after uracil has flipped out from the DNA backbone rather than “pushing” uracil from the minor groove and thus contributes to efficient catalysis by increasing the lifetime of the extrahelical base (58).

### Lysine

Following in the footsteps of Toney and Kirsch (8), several groups have since demonstrated the potential to rescue lysine mutants with bases. Lys80 in leucine dehydrogenase, an NAD(P)^+^-dependent oxidoreductase, has been mutated to Ala, Gln, and Arg to understand the role of lysine in catalysis (59). One M ammonia restored the deamination activity of Lys80-mutant leucine dehydrogenase from 1.5% to 20% that of WT leucine dehydrogenase (59). Satisfyingly, K80Q was rescued to a lesser extent with ammonia than K80A, whereas K80R was nearly unaffected by ammonia, suggesting the importance of sterics in chemical rescue strategy design. A Brønsted plot was generated against a handful of primary amines to show that basicity and molecular volume together determine the effectiveness of activity rescue (59). Enzymatic characterizations made possible by site-directed mutagenesis and the addition of exogenous amines helped unravel the mechanism through which the lysine ε-NH2 facilitates the nucleophilic attack by water on the substrate α-carbon (59). Similarly, small molecules have been used to restore biochemical functions in various protein classes including rhodopsin (60), tryptophan synthase (61), alanine racemase (62), and isochorismate-pyruvate lyase (63).

When lysine is mutated into cysteine, chemical rescue can be facilitated by covalent modifications, as alluded to in Figure 2. Installing a K258C mutation in aspartate aminotransferase rendered the enzyme inactive. The introduced Cys was amenable to aminoethylation by 2-bromoethylamine, which restored enzymatic activity to 7% of WT aspartate aminotransferase (64). A similar approach was also applied to *Bacillus stearothermophilus* pyruvate kinase to study the importance of Lys221 in catalysis. Installing C9S/C268S/K221C mutations rendered the enzyme nearly inactive while leaving K221C as the only cysteine residue, and aminoethylation of this Cys by N-[2-iodoethyl]trifluoroacetamide rescued the triple-mutant enzyme to 4.5% of the WT pyruvate kinase activity (65). This method, while effective, is restricted by the highly optimized conditions required for successful aminoethylation, which involves stepwise protection or mutation of off-target Cys residues coupled with unfolding and refolding of the enzyme (64, 65).

Besides unmodified lysine residues in enzyme active sites, many lysines are posttranslationally modified to modulate enzymatic function. In the amidohydrolase allantoinase (ALLase), for example, Lys146 is often carboxylated to promote binuclear metal center self-assembly and facilitate catalysis (66, 67). To restore the enzymatic activity of the carboxylated lysine, short-chain carboxylic acids were added to the K146A-mutant ALLase (68). This approach raised mutant catalytic activity from 1 to 6% to ∼10% of WT ALLase. Despite the modest activation achieved by the exogenous reagents, this study showcased the prospect of using chemical rescue to study complicated catalytic networks such as those in metalloenzymes.

### Tryptophan

While there have not been many examples of chemical rescue on tryptophan mutations, an elegant study by Karanicolas et al. in 2012 first demonstrated the feasibility of using small hydrophobic ligands to fill in enzyme cavities (69). When Trp33 in *S. solfataricus* β-glycosidase (β-gly) was mutated into Gly, a 730-fold decrease in catalytic efficiency was observed. Leveraging structural biology, the authors pinpointed this Trp as a neighboring residue to the β-gly active site. The W33G mutation created a cavity that caused the active site residue Trp433 to change its orientation, thereby breaking substrate coordination and abolishing catalysis. Exogenous indole fully rescued β-gly by filling the cavity and preventing Trp433 from shifting over. Notably, this strategy was successfully reproduced in live *E. coli* cells. A similar approach was applied to the rescue of a W492G mutant *E. coli* β-glucuronidase (69).

In 2013, through a combination of computational predictions and experimental validations, the same group followed up on their previous study to systematically explore the generalizability of chemical rescue on cavity-inducing mutations (70). Reporter assays and structural analyses on *E. coli* homodimeric proteins (YeaZ, Orn, and TadA) carrying Trp to Gly mutations, as well as biochemical studies on +5 GFP and β-gluc, suggest that indoles can also salvage Trp mutations that induce global misfolding and destabilization of proteins.

### Tyrosine

Ketosteroid isomerase is a bacterial enzyme that shifts a double bond into conjugation with the 3-ketone in the A-ring of steroids (71). It was shown that a key tyrosine, Tyr14, now known to stabilize the enolate intermediate by serving as a hydrogen bond donor to the 3-ketone, is very mutationally sensitive. The activity of the Y14G-mutant ketosteroid isomerase could be rescued by about 10-fold over baseline by 2 mM phenol, though the rescued enzyme remained well below 1% of WT activity (71).

In xylose reductase, a fungal NADH-dependent enzyme that interconverts an alcohol and ketone, Tyr51 is important for catalysis. Y51A-mutant xylose reductase is partially rescued by a bromide ion, which mimics the phenolate ion to facilitate the NAD^+^-dependent oxidation of xylitol (72).

TbtD is a *Thermobispora bispora* pyridine synthase that forms a pyridine from two dehydroalanine moieties as part of the production of thiomuracin, a bioactive, posttranslationally modified thiopeptide (73). Y319 was found to play a key role in catalysis, as Y319F and Y319A mutants of TbtD accumulated a dearomatized reaction intermediate but produced much less of the final pyridine product. The addition of 10 mM phenol to Y319A TbtD led to a substantial increase in pyridine formation. Further, the use of various phenol derivatives showed that rescue compounds containing electron-withdrawing groups, which decrease the hydroxyl p*K*_a_, promoted product formation, while compounds with electron-donating groups and higher p*K*_a_ values caused increased intermediate accumulation. Together, these results suggest that Y319 likely plays dual, opposing roles in the two reaction steps, though steric effects associated with the rescue compounds could also influence the relative amounts of intermediate and product that are formed (73).

## Chemical rescue for biological phenotypic and pathway analysis

Chemical rescue of mutant proteins in living cells can provide key functional information about the protein of interest and the pathway(s) in which it participates. Compared to transient transfection and gene knockdown approaches that modulate protein expression on timescales ranging from days to the entire lifetime of a cell or model organism, chemical complementation of a loss-of-function mutation can be achieved rapidly, within 1 to 10 min (12, 74, 75). Thus, chemical rescue is well-suited to provide information about specific “early” cellular events that could be masked by chronic changes in systems perturbed by classical genetic approaches. On the other hand, while small molecule inhibitors that can provide time-resolved pharmacological functional data points are available for many proteins, switching “off” a protein may produce distinct impacts compared to switching it “on.” For cell surface receptors like receptor tyrosine kinases, natural agonists that bind the ectodomain and promote dimerization can provide rapid activation and kinetic insights into cellular phosphorylation events. Such cell-permeable agonists are not typically available for intracellular proteins. In this way, chemical triggers for an intracellular protein engineered to be inactive but rescuable may facilitate the acquisition of similar information.

Requirements for the successful chemical rescue of mutant proteins in live cell studies include the following: 1) a large dynamic range between the baseline mutant activity and the stimulated activity, 2) relatively low toxicity and high cell permeability of the small molecule rescue agent, and 3) preservation of the physiological characteristics of the complemented protein. For example, if the rescued mutant protein is an enzyme, the catalytic parameters of the complemented mutant should match those of the native enzyme as closely as possible to preserve natural substrate selectivity. However, functional rescue in cells can be observed even when the rescued enzyme falls significantly below WT catalytic levels in some cases, as was observed for Csk (11). This result may occur because the full activity of the native enzyme is much more than needed to achieve a cellular effect. In such instances where rescued activity is still well below the WT level, a large dynamic range between unrescued and rescued enzyme is still likely to be critical. Ideally, evaluation of the rescue characteristics can initially be assessed with purified mutant proteins using *in vitro* assays. However, it is not always straightforward to generate such purified proteins, so validation experiments in cell extracts or intact cells are sometimes employed.

### Chemical rescue of enzyme point mutations

As detailed above in our discussion of chemical rescue for enzyme mechanism analysis, imidazole has been shown to complement protein tyrosine kinases with active site Arg mutations. PTKs are important cell signaling enzymes that participate in controlling virtually every aspect of cellular life. Thus far, chemical rescue has been applied to Csk, Src, and Abl kinases in cellular systems (11, 12, 74, 75). These proteins are all nonreceptor PTKs and thus cannot be activated directly by exogenous ligands, but imidazole can rescue the activity of Arg to Ala mutants by > 100 fold. Imidazole has a p*K*_a_ of approximately 7 and is quite cell-permeable in its neutral form. The imidazolium ion is likely the active form of the rescue agent (25), and a substantial fraction of imidazole is likely to be protonated in the cytosol or nucleus at physiological pH. The potency of imidazole tyrosine kinase rescue is modest with an EC_50_ of ∼5 mM, but imidazole itself appears to be relatively nontoxic, especially during short time frames during which rescue technology is typically applied.

Of the tyrosine kinases investigated, Src has been the most widely studied by chemical rescue with imidazole. Demonstration of the technique was originally performed with v-Src using mouse embryonic fibroblasts in which the three major Src family members—Src, Yes, and Fyn—were genetically ablated (SYF MEF cells) (12). v-Src is an oncogenic, constitutively active enzyme lacking the C-terminal phosphorylation found in the proto-oncogene c-Src that renders the latter form of this enzyme catalytically quiescent. SYF MEF cells were stably transfected with R388A v-Src or a negative control catalytic base mutant (D386N) that was shown to be unrescuable *in vitro.* Tyrosine phosphorylation of the transfected Src and other proteins was assessed in response to imidazole treatment. Only R388A v-Src showed imidazole-induced tyrosine phosphorylation, which was also rapidly reversible upon imidazole washout. Using a FRET-based cell sensor, v-Src chemical rescue was imaged in live cells, and tyrosine phosphorylation was observed within 1 min of imidazole treatment. Chemical rescue also provided the surprising insight that the R388A mutant of c-Src showed robust tyrosine phosphorylation activity after stimulation with imidazole, even though c-Src was long considered to be autoinhibited under normal cellular conditions (12). These results provide evidence for the importance of the tyrosine phosphatases that counteract constitutive c-Src activity and thereby render it invisible under basal conditions.

Analysis of mutant tyrosine kinase chemical rescue using mass spectrometric–based phosphotyrosine proteomics has identified a plethora of novel targets of both Src and Abl (74, 75). A number of these putative substrates were validated using IP-western blot analysis, and some were further examined in the context of growth factor stimulation and downstream signaling networks. Chemical rescue helped to characterize a role for Src in Akt activation in the context of angiogenesis (76). In addition, chemical rescue was applied to confirm an unexpected function of Src in p53 tyrosine phosphorylation (77) and in Toll-like receptor regulation of inflammation (78). In addition, chemical rescue of mutant Csk has provided insights into the role of Csk in the formation of stress fibers and focal adhesions (11).

Beyond protein kinases, a mutant Ser protease has also been chemically rescued in cells (79). The cytomegalovirus protease assemblin contains an atypical Ser-His-His catalytic triad. Like many proteases, assemblin is generated from a longer precursor by autoproteolysis. Mutating the His proximal to the Ser to Gly knocked out assemblin's proteolytic activity, but this loss of activity could be complemented efficiently with imidazole *in vitro* and in mammalian cells. Notably, the full-length assemblin precursor was not complemented in this fashion. This differential rescue activity provides a tool to study specific forms of the cytomegalovirus protease *in vivo* and tease apart their functions.

More recently, a class of mutant multiprotein cellular filament assemblies in yeast called septins was elegantly complemented by treatment with guanidinium (80). While guanidinium is perhaps best known as a denaturant, in this context, it is proposed to substitute for an Arg sidechain that was “lost” during yeast evolution. Using such chemical rescue, the investigators were able to uncover aspects of the complex molecular processes driving septin assembly. Motivated by these interesting findings, the group undertook further screening studies to identify other yeast proteins that could be rescued chemically. Although guanidinium, trimethylamine-N-oxide, and dimethyl sulfoxide were able to rescue a few rationally designed proteins as well as mutants in an untargeted temperature-sensitive library, the extent of rescue appeared weak *in vivo*. Moreover, in general, these studies were unable to pinpoint whether the rescue mechanism was specific to particular proteins or occurred through a more general effect on protein folding and stability (81).

### Enzyme activation using domain engineering

Several elegant methods for the chemical control of enzymatic activity using large structural mutations have been employed. In one approach, genetic insertion of the protein FKBP into the kinase catalytic domain leads to a loss of cellular function that can be rescued by adding rapamycin or rapamycin analogs to the cell culture media (15). In another case, the N-lobe of the kinase domain is genetically split, inactivating the enzyme. Rescue is achieved by an FKBP/FRB dimerization event achieved with rapamycin analog (82). Using these approaches on Src (83, 84) and Abl (85) has led to novel insights into the cell signaling functions and substrate targets of these kinases.

These methods and related split protein approaches have been further applied to other enzymatic classes. Inducible gene-editing systems have been developed by splitting the nuclease Cas9 into N- and C-terminal fragments, which are fused to FRB and FKBP respectively and enable reconstitution of the active enzyme in the presence of rapamycin (86). Ligand-responsive domain engineering was also used to rescue the function of the p300 histone acetyltransferase. It had previously been shown that deletion of the C-terminal tail of the p300 HAT domain abolishes its catalytic activity (87). By using a conditional split intein, which, when bridged by the small molecule rapamycin, can splice the p300 pieces back together, cellular restoration of catalytic activity could be obtained (88).

In addition to small molecules, light has also been employed to induce enzymatic activity. In one case, two copies of an engineered photodissociable protein domain were inserted at the kinase N-terminus and within a loop in the C-lobe of the kinase, forming a dimer that blocked kinase activity (89). The dimer dissociated upon exposure to 500 nm light, activating the kinase, and re-dimerized upon exposure to 400 nm light to halt kinase activity. This approach was used to study signaling downstream of the kinases MEK1, MEK2, and Raf1 (89). In another case, a constitutively active mutant form of the Rho GTPase Rac1 was fused to the phototropin light oxygen voltage domain, blocking Rac1 from binding to its effectors until exposure to 458 nm or 473 nm light induced a conformational change in the light oxygen voltage domain that activated Rac1 (90).

### Rescue of caged enzymes

**Figure 6:**
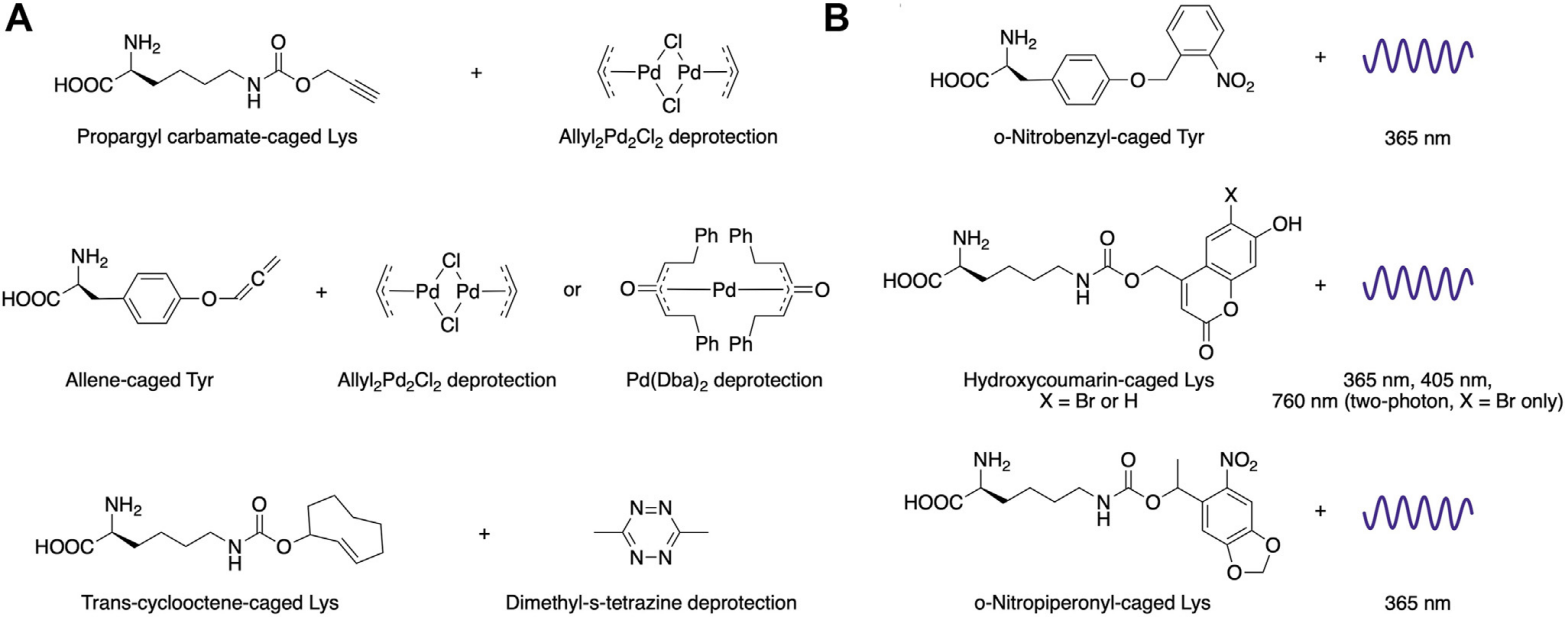
**Non-natural caged amino acids.** Structures of common (*A*) chemically cleavable and (*B*) photocleavable caged amino acids (14, 91, 92, 93, 94, 95, 96, 97, 98).

Unnatural amino acid mutagenesis by nonsense suppression has become a powerful method for the analysis of proteins *in vitro* and in living cells. One set of unnatural residues involves protecting the sidechains with blocking groups that perturb their natural functions. Chemical functionalities used in this context include Pd-removable propargyl carbamate groups on Lys (91) and allenes on Tyr (92) as well as tetrazine removal of a trans-cyclooctene–modified Lys (93). The incorporation of o-nitrobenzyl-Tyr (94, 95), hydroxycoumarin-protected Lys (96), and o-nitropiperonyl protected Lys (14, 97) *via* nonsense suppression mutagenesis followed by optical uncaging offers spatiotemporal control of cellular enzymes for the study of their downstream activity (Fig. 6). While o-nitrobenzyl-Tyr and o-nitropiperonyl protected Lys are cleaved most efficiently by 365 nm light, hydroxycoumarin-protected Lys can be cleaved at both 365 nm and 405 nm, allowing for the orthogonal use of multiple photocleavable residues (14, 94, 95, 96, 97, 98). Further, bromohydroxycoumarin-caged Lys is efficiently cleaved not only by single-photon excitation at 365 nm and 405 nm but also by two-photon excitation at 760 nm, providing further orthogonality and enabling the use of longer wavelengths for photocleavage (98).

## Chemical rescue for disease therapy

The potential for chemical rescue of mutant proteins as therapeutics has been described for enzymes, membrane transporter and receptor proteins, nuclear hormone receptors, and transcription factors. There are myriad genetic diseases involving germline point mutations that result in loss of function of the affected protein. In principle, such amino acid replacements could lead to the loss of an active site catalytic residue sidechain or directly disrupt a binding event. Most commonly, however, these loss-of-function missense mutations destabilize the native fold of the protein. Such destabilizing point mutations can lead to protein aggregation and/or target the misfolded protein for proteasomal degradation. For membrane or secreted proteins, destabilizing mutations can prevent proper cellular trafficking of the protein through the endoplasmic reticulum, Golgi body, or plasma membrane, preventing normal cellular function.

**Figure 7:**
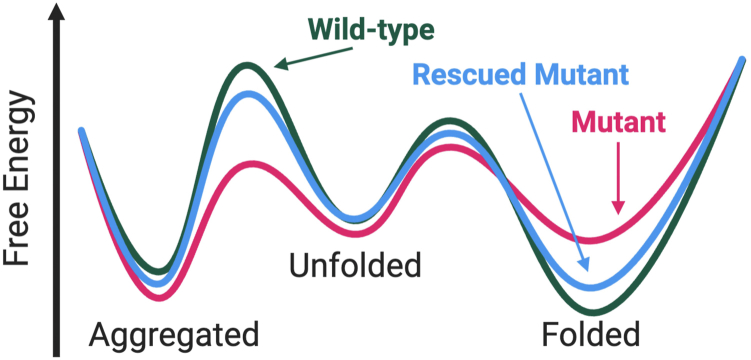
**Chemical rescue through protein stabilization.** Thermodynamic comparison of WT or mutant protein folding in the presence or absence of a stabilizing ligand. Created in BioRender. O, T. (2025, https://BioRender.com/f4oz944).

At first thought, one might imagine that it would be extremely challenging to identify a small molecule agent that can bind to the mutant protein precisely in the vicinity of the altered amino acid residue and stabilize the structure. However, a requirement for the rescue agent to bind to the affected protein near the mutant residue is likely unnecessary for restoring structural stability. Rather, the small molecule rescue agent could interact with a complementary surface remote from the site of mutation if the conformation stabilized by the compound preferentially corresponds to the native structure. This effect can be readily understood using a simple thermodynamic analysis (Fig. 7). If a stabilizing ligand binds more tightly to the folded structure *versus* the non-native state of the mutant protein, then the ligand should effectively stabilize the natively folded protein structure. Because these thermodynamic effects only depend on global stabilization of the native folded state, understanding where or how the small molecule interacts with the mutant protein to rescue its native folding is not essential to having a pharmacologically effective tool.

### Enzymes

There are many well-characterized genetic diseases in which the defective protein is a key enzyme involved in amino acid or carbohydrate metabolism. Cystathionine-β-synthase (CBS) is an enzyme that catalyzes the formation of cystathionine from homocysteine and serine as part of the methionine catabolic pathway. Loss-of-function mutations in CBS result in the buildup of homocysteine, which can cause developmental neurological and skeletal pathologies in affected individuals. I278T CBS is one such mutation that leads to diminished formation of the active tetramer form of CBS (99, 100). This mutant can be partially rescued by chemical chaperones such as dimethyl sulfoxide or sorbitol (101).

Phenylketonuria is a relatively common genetic disease that results from loss-of-function mutations in phenylalanine hydroxylase (PAH). Several PAH point mutants distributed throughout the sequence result in a misfolded enzyme that cannot assemble into its normal tetrameric state. Phenylketonuria patients have cognitive developmental problems that stem from elevated phenylalanine levels. The PAH cofactor tetrahydrobiopterin has been shown to stabilize mutant PAH, and treatment with excess tetrahydrobiopterin has been shown to rescue mutant forms of PAH through a combination of PAH stabilization and enhancement of enzyme kinetics (102, 103). A pharmacological screen identified the dihydroisoquinoline compound III as a cell active stabilizer of the physiological PAH structure of the mutant proteins (104).

Fabry's disease results from α-galactosidase A (GalA) mutations, and patients with Fabry's suffer from renal failure and cardiovascular events. Pathological mutations that destabilize GalA and cause its aggregation in the endoplasmic reticulum can somewhat paradoxically be complemented by treatment with deoxygalactonojirimycin (DGJ), which is a competitive inhibitor of GalA (105). The binding of DGJ was found to stabilize mutant GalA, preventing its aggregation in the endoplasmic reticulum and allowing it to reach the lysosome, where DGJ dissociates and leaves behind a functional GalA enzyme (105). Related studies have been carried out on destabilized N370S-mutant β-glucosidase found in Gaucher's disease, a heavily studied genetic disorder that is associated with multiorgan pathophysiology. As seen with DGJ for GalA, the catalytic inhibitor N-(n-nonyl)deoxynojirimycin shows promise in promoting the stability of the mutant β-glucosidase and facilitating its trafficking to the lysosome (106). Other disease-associated destabilized mutant enzymes that have shown some promise for rescue through chemical complementation include galactocerebrosidase in Krabbe disease, which can be rescued by α-lobeline and 3′,4′,7-trihydroxyisoflavone, (107), tripeptidyl peptidase I in CLN2 disease, one form of which was partially rescued by DMSO (108), and HDAC8 in Cornelia de Lange Syndrome, multiple forms of which are partially or fully rescued by *N*-(phenylcarbamothioyl)-benzamide (109).

### Membrane proteins

Genetic diseases involving mutant membrane or secreted proteins can often prevent the proper trafficking of the misfolded protein from making its way to its normal cellular site. The most famous example along these lines is the cystic fibrosis transporter regulator (CFTR) chloride ion channel. Cystic fibrosis is one of the most common autosomal recessive genetic diseases in the United States and is characterized by a defect in epithelial cell fluid transport that leads to frequent pneumonia and pancreatic disorders. The most common pathogenic mutations in CFTR, including the ΔF508 mutation, lead to protein instability and prevent it from reaching the plasma membrane, where it performs its transport function (110). Ivacaftor is a synthetic small molecule that works to stabilize some mutant forms of CFTR and enhances their presence and transport function on epithelial cell membranes (111). The most recent formulation for cystic fibrosis combines ivacaftor with another folding corrector, elexacaftor, and a channel potentiator, tezacaftor, and has shown efficacy toward CFTR mutants found in over 90% of cystic fibrosis patients (112, 113). Beyond CFTR, advances in the chemical rescue of disease-associated mutant membrane proteins include aquaporin-2, the HERG K channel, the GnRH receptor, and others (Table 2).

**Table 2:** Summary of membrane protein rescue examples

Target protein	Protein function	Associated disease	Selected rescue agents
CFTR	Membrane Cl^–^ ion channel	Cystic fibrosis	Ivacaftor/elexacaftor/tezacaftor combination therapy (112, 113)
Aquaporin-2	Membrane water channel	Nephrogenic diabetes insipidus	Glycerol (128)
hERG	Membrane K^+^ ion channel	Long QT syndrome, a type of cardiac arrythmia	E-4031, astemizole, cisapride, glycerol (129), terfenadine, fexofenadine (130), thapsigargin (131)
GnRH receptor	Hormone receptor	Congenital hypogonadotropic hypogonadism (Kallmann’s syndrome)	IN3 (132), IN30, IN31B, Q76, Q89, A-177775, A-222509 (133), SR-01000211348-2, SR-01000439482-2 (134)
Vasopressin V2 receptor	Hormone receptor	Nephrogenic diabetes insipidus	SR121463A (135), YM087 (conivaptan) (136), SR121463B (satavaptan), VPA985 (lixivaptan) (137), SR49059 (relcovaptan), OPC31260 (mozavaptan), OPC41061 (tolvaptan) (138), WAY-151932, OPC23h, MCF57 (139)
Rhodopsin	G-protein coupled receptor	Rhodopsin-mediated retinitis pigmentosa	11-*cis*-retinal (140), metformin (141), S-RS1 (142), chromenone derivatives (143), quercetin (144)
MCT8	Thyroid hormone transporter	Allan-Herndon-Dudley syndrome	Sodium phenylbutyrate, genistein (145)
Munc18-1	Synaptic complex subunit	Epilepsy, neurodegenerative disorders, intellectual disability, movement disorders	4-phenylbutyrate, sorbitol, trehalose (146, 147)

### Transcription factors

The tumor suppressor p53 is one of the most commonly mutated genes in human cancer. Functioning as a key DNA-binding transcription factor that is activated by DNA damage, p53 protein stimulates genes involved in arresting the cell cycle, like p21, and apoptotic pathways, such as PUMA (114). Mutations in the p53 DNA-binding domain or other parts of the protein commonly lead to its destabilization. An early attempt at screening for small molecules that could stabilize mutant p53 led to the identification of the quinazoline derivative CP-31398 (115). While CP-31398 appeared to show promise in stabilizing mutant p53 and even shrunk tumors in mouse xenograft studies, subsequent studies revealed that CP-31398 likely works by binding DNA rather than p53 protein (116). A renewed effort to find *bona fide* mutant p53 stabilizers has continued and has employed more robust binding and structural methods to characterize mutant p53-small molecule interactions. In two recent studies, a series of carbazole derivatives have been shown to bind to a protein cavity in proximity to mutated Y220C or Y220S of p53 (117, 118).

Nuclear hormone receptors, including the thyroid hormone and vitamin D receptors, regulate transcription by binding to DNA in enhancer elements upon the binding of their natural small molecule ligands. Disease-associated mutations in the thyroid hormone and vitamin D receptors reduce the affinities of these receptors for their natural ligands. Synthetic analogs of these ligands have been developed that can complement these mutations and offer a pathway for treating patients with nuclear hormone receptor defects (119, 120, 121, 122, 123).

## Future perspectives and challenges

Chemical rescue has emerged as a powerful approach for both enzymological studies and the development of novel therapeutics. The simplest form of chemical rescue, in which a single amino acid mutation inactivates the protein of interest while introducing a binding site for a chemically complementary small molecule, allows not only the assessment of the functions of specific amino acids in catalysis but also rapid and reversible control over enzymatic activity.

Despite the promise of this method, chemical rescue of point mutants is limited by the low affinity of many rescue compounds for the target of interest. While millimolar concentrations of rescue compounds are tolerated for *in vitro* enzymatic studies, such high concentrations could introduce off-target effects or toxicity in cellular or organismic systems. This limitation has been overcome in cells by relatively nontoxic rescue compounds such as imidazole (12, 74, 75), but this approach only works for certain compound/enzyme combinations.

To enable the broader application of cellular chemical rescue methods, two possible strategies can be employed or combined: 1) the creation of a larger binding pocket through combinatorial mutation or 2) the introduction of nucleophilic amino acids to bind covalently to rescue compounds at lower concentrations. While these approaches may limit the generalizability of a specific set of chemical rescue mutations across a class of proteins, they may help overcome the low potency of existing rescue compounds by taking advantage of the larger binding pocket to form higher-affinity interactions or by using covalent bonding to enhance the effective binding affinity. In this regard, recent advances in artificial intelligence methods capable of accurately predicting protein–ligand interactions have high potential to aid in the rational design of protein-rescue agent pairs (124, 125).

Although alternative methods such as the introduction of large protein domains or the caging of individual amino acids have also been employed, this mutational approach can better retain the endogenous protein structure and protein–protein interactions while avoiding the need for genetic code expansion. In addition, mutation-based approaches can be implemented as germline knock-in edits to the protein of interest, allowing for the expression of the mutant protein under the control of endogenous gene expression regulation. As a result, chemical rescue can be applied to the protein of interest at physiologically relevant expression levels and in only the relevant subcellular compartments, provided that the introduced mutations do not structurally alter the rescuable mutant enzymes.

In addition to various methodological improvements, the future of chemical rescue should also include its application toward a wider range of enzyme classes in living systems. While small-molecule chemical rescue has been applied to the cellular study of Tyr kinases, the protease assemblin, and yeast septins, it has yet to be implemented for Ser/Thr kinases, most proteases, and a variety of other enzyme classes. Although alternative approaches such as caging or domain engineering have also emerged for the study of these proteins, a robust small-molecule approach will complement these methods by enabling the study of enzyme-substrate preferences using a more native form of the protein of interest.

Beyond basic biology, chemical rescue of misfolded proteins has emerged as a promising therapeutic approach, particularly for the treatment of cystic fibrosis. Further efforts to identify and develop potent small-molecule binders that stabilize the native conformations of disease-relevant mutant proteins could represent a major area for therapeutic development. Following the lead of ivacaftor, elexacaftor, and tezacaftor, this area of research could provide substantial benefit to patients with unmet needs, extending chemical rescue beyond the bench and into the clinic. Further, new computational methods based on deep learning and neural networks may facilitate the identification of lead compounds that rescue disease mutants well beyond what has been achievable with previous *in silico* and *in vitro* methodologies.

Overall, then, chemical rescue has served as a foundational method in enzymology for the characterization of enzyme mechanisms and kinetics since its development in the 1970s and 1980s. Building upon this work, further efforts to expand, enhance, and elaborate upon chemical rescue for the study of protein function and the correction of protein dysfunction will continue to play a key role in biochemistry and chemical biology in the years to come.

## Conflicts of interest

The authors declare that they do not have conflicts of interest with the contents of this article.
